# Flooding with shallow water promotes the invasiveness of *Mikania micrantha*

**DOI:** 10.1002/ece3.5465

**Published:** 2019-07-21

**Authors:** Maofeng Yue, Hanxia Yu, Weihua Li, Aiguo Yin, Ye Cui, Xingshan Tian

**Affiliations:** ^1^ School of Biological and Food Engineering Guangdong University of Petrochemical Technology Maoming China; ^2^ Institute of Ecological Science, Guangdong Provincial Key Laboratory of Biotechnology for Plant Development, Guangzhou Key Laboratory of Subtropical Biodiversity and Biomonitoring, School of Life Science South China Normal University Guangzhou China; ^3^ Institute of Plant Protection Guangdong Academy of Agricultural Sciences/Guangdong Provincial Key Laboratory of High Technology for Plant Protection Guangzhou China

**Keywords:** germination rate, invasive species, population size, seedling growth, water conditions

## Abstract

The invasive ability of alien plants is not only affected by their biological characteristics but also by environmental factors. Therefore, investigating the relationship between plant growth and environmental factors is helpful for predicting the invasive potential of alien species. *Mikania micrantha* H.B.K. (a vine of Asteraceae) is one of the top 10 most invasive weeds worldwide and causes serious damage to agroforestry ecosystems. Water is an important environmental factor that affects plant growth; however, the relationship between water conditions and the rapid growth of *M. micrantha* is not clear. In this study, 162 *M. micrantha* population sizes were investigated in dry, wet and aquatic habitats in the Pearl River Delta region of Guangdong, China. In addition, the seed germination and seedling growth characteristics of *M. micrantha* were determined by submerging tests. The results showed that the population size of *M. micrantha* was the largest in aquatic habitats, and the soil moisture content was positively correlated to the population size in dry and wet habitats. Furthermore, *M. micrantha* seeds could germinate underwater and grow out of the water surface at a depth of 6 cm with a survival rate of 7.4%. Aquatic habitat promoted vine elongation, whereas dry habitats resulted in the reverse pattern. After 8 weeks of water treatments, the vine stem length was 2 and 3 times longer in the aquatic habitat than the wet and dry habitats, respectively. The total root length, root volume, and root tip number increased significantly in the aquatic habitat when compared to those in the wet habitat; however, these parameters exhibited the opposite pattern in the dry habitat. The results showed that flooding with shallow water is conducive to the invasiveness of *M. micrantha*, suggesting that water is the key determinant during the intrusion process of *M. micrantha* populations.

**Open Research Badges:**

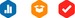

This article has been awarded Open Data, Open Materials and Preregistered research design Badges. All materials and data are publicly accessible via the Open Science Framework at https://osf.io/ksz2f/?viewonly=30b6fec21f0447edbdfc9cebe2b01065, https://osf.io/a5ymf/ and https://osf.io/ksz2fl?viewonly=cfcbfOfc829c402fb22deb3be801dffc

## 1 INTRODUCTION

Biological invasion represents the second most important threat to global biodiversity after land use change (Chytrý et al., 2012). The damage caused by invasive plants has increased annually and resulted in serious threats to human health, agricultural production, and biodiversity around the world (Ozaslan et al., 2016; Pejchar & Mooney, 2009). During the intrusion process of invasive plants, environmental factors play an important role on the growth and distribution of invasive plants (Ensslin, Mollel, Hemp, & Fischer, 2018; Karkanis, Ntatsi, Alemardan, Petropoulos, & Bilalis, 2019). Therefore, investigating the relationship between plant growth and environment factors is helpful for predicting the invasive potential of alien species and establishing effective management strategies.

Soil moisture is a key environmental factor for plant growth and development. The effect of soil moisture on invasive plants has received widespread attention, especially in the context of global climate change (Awada et al., 2019; Saptiningsih, Dewi, Santosa, & Purwestri, 2018). Different invasive plants showed different responses to soil water conditions; for example, the invasive species *Centaurea diffusa* Lam. and *Poa pratensis* L. showed strong adaptability to drought, which leads them to form dominant populations in arid environments (Dong, Patton, Wang, Nyren, & Peterson, 2014; Turner, Nurkowski, & Rieseberg, 2017), whereas the invasive plants *Bidens pilosa* L. and *Alternanthera philoxeroides* (Mart.) Griseb. showed stronger tolerance to waterlogging than natives, which benefits their invasion ability in waterlogged environments (Chen, Zhou, Yin, Liu, & Luo, 2013; Yue et al., 2019). Thus, the response of invasive plants to soil moisture will be helpful for determining the habitats that are more vulnerable to invasion.

*Mikania micrantha* H.B.K. (belonging to the family Asteraceae) is a fast‐growing perennial herbaceous or slightly woody vine native to tropical America (Lowe, Browne, Boudjelans, & De Poorter, 2001) and has been listed as one of the top 10 most invasive weeds worldwide (Holm, Plucknett, Pancho, & Herberger, 1977). This species has rapidly expanded in tropical and subtropical parts of the Asia‐Pacific region, which has resulted in serious damage to plantation crops and agroforestry ecosystems (Day et al., 2016). The vine stem grows rapidly and can increase by 20 cm in one night (Du, Yang, Li, & Yin, 2006); therefore, the plant has been nicknamed “mile‐a‐minute” weed (Waterhouse, 1994). Due to the rapid elongation of its vine stems, *M. micrantha* can climb over other plants and prevent them from receiving adequate sunshine, thereby hindering their growth and reproduction or eventually causing their death (Huang et al., 2000; Zhang, Ye, Cao, & Feng, 2004); however, little is known about how such rapid growth is maintained.

Although *M. micrantha* is found in various habitats, such as disturbed roadsides, wastelands, plantations and secondary forests in low‐altitude valleys, barren farmlands, and orchards, as well as the sides of ditches and rivers (Zhang et al., 2004), some studies have shown that the species prefers humid environments (Murphy et al., 2013; Shen et al., 2015). However, the effect of soil moisture on its population size has rarely been addressed (Xu, Shen, Zhang, Li, & Zhang, 2013).

Here, we hypothesize that *M. micrantha* presents a number of biological characteristics to adapt to aquatic or wet habitats, thereby benefiting its population expansion. Firstly, we investigated the population size of the invasive species *M. micrantha* under three water conditions (dry habitats, wet habitats, and aquatic habitats). Second, the seed germination and seedling growth characteristics were determined by different submerging tests to further elucidate the specific effects of water conditions. Lastly, the survival rates of *M. micrantha* were examined at different water‐drown depths to reveal the effects of flooding. Our aim is to provide a scientific basis for predicting the invisibility or susceptibility of ecosystem and setting up an effective management strategy for *M. micrantha* in the field.

## 2 MATERIALS AND METHODS

### 2.1 Population sizes of *M. micrantha*

**Figure 1:**
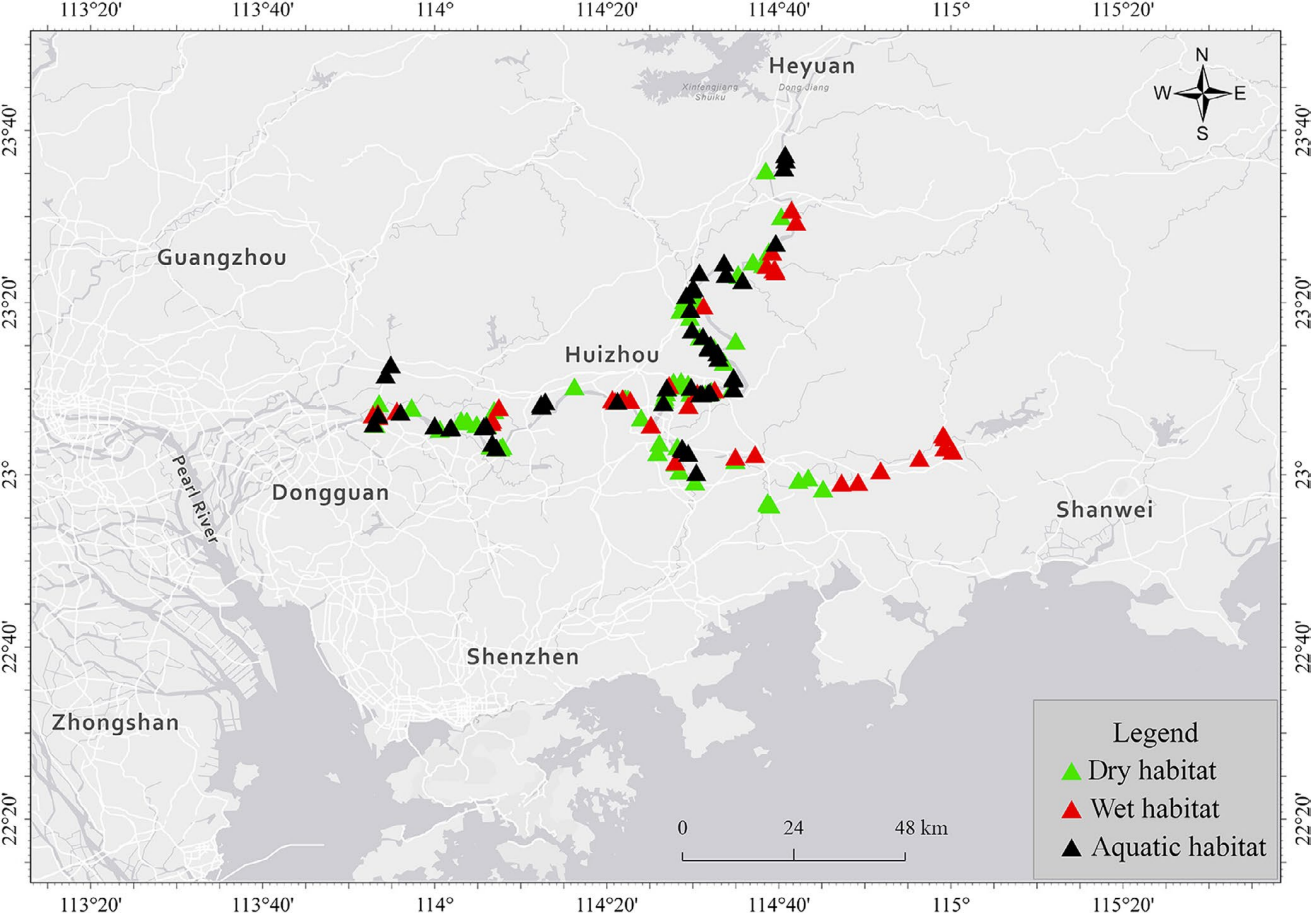
The locations of 162 *Mikania micrantha* populations in drylands (dry habitats; *n* = 74), on riverbanks (wet habitats; *n* = 36), and in wetlands with shallow water (aquatic habitats; *n* = 52) in the Pearl River Delta region of Guangdong, China

The *M. micrantha* population sizes were randomly investigated in the field across agricultural systems in the Pearl River Delta in Guangdong, China in April 2015 (Figure 1). When a population appeared continuously in one habitat, it was treated as one population, and when the gap between populations was >300 m, it was considered two habitats. We investigated the *M. micrantha* population size in drylands, on riverbanks, and in wetlands by approximating their geometric shape using a measuring tape in the field. The soils of the *M. micrantha* populations in drylands and on riverbanks were brought back to the laboratory to determine their relative humidity (Moore, 2001). Soils with a relative humidity of <60% were considered to be from dry habitats, and those with a relative humidity of more than 80% but not covered by water were considered to be from wet habitats (Burns, 2004; Klemedtsson, Svensson, & Rosswall, 1988). The wetlands with a water depth of no more than 30 cm were regarded as aquatic habitats. The dry habitats included wastelands, roadsides, orchards, and secondary forests, and the wet habitats included both sides of river banks where water flow could be seen with the naked eye. The aquatic habitats included ditches, ponds, and depressions where the water was almost stationary. A total of 162 *M. micrantha* populations were investigated in three different habitats, including 74 populations in dry habitats, 36 in wet habitats, and 52 in aquatic habitats.

### 2.2 Seed germination of *M. micrantha* under different water conditions

To study the effects of drought on the seed germination of *M. micrantha*, drought was imitated by a polyethylene glycol 6000 (PEG6000) solution, with 0, 85.4, 160.6, 212.6, 255.0, or 291.7 g of PEG6000 dissolved in 1 L of distilled water to obtain aqueous solutions with osmotic potentials of 0, −0.1, −0.3, −0.5, −0.7, or −0.9 MPa, respectively, according to Wei et al. (2009). Germination tests were conducted by placing 30 seeds in a 9‐cm‐diameter Petri dish containing two layers of filter paper, which were moistened with 8 ml of distilled water or PEG6000 solution. In addition, Petri dishes containing *M. micrantha* seeds were placed at the bottom of transparent plastic boxes with a water depth of 6 cm to test the effects of flooding on seed germination. All Petri dishes used in this experiment were placed in a growth chamber (RXZ‐430c, Ningbo Southeast Instrument Co., Ltd) at 30/25°C (day/night) under a 12‐hr photoperiod. Each treatment set consisted of eight replicates. Germinated seeds were counted after 14 days, and the seed germination rates under the different treatments were calculated (Wei et al., 2009).

### 2.3 Growth of *M. micrantha* seedlings under different water‐submerging test

The experiment was conducted in Guangzhou (E 113°21′, N 23°9′; elevation, 14 m), Guangdong Province, China from April to June 2016. Seedlings of similar sizes were transplanted to plastic pots with sandy soil (pH, 6.0; soil organic C, 9.50 g/kg; available N, 59.63 mg/kg; available P, 40.5 mg/kg; and available K, 44.5 mg/kg; the same soil was used in the subsequent experiment) at the 2‐leaf stage. Water treatments were applied when the seedlings were at the 6‐leaf stage. Three water treatments were established: soil with a 40% water‐holding capacity as the dry habitat; soil with a 90% water‐holding capacity as the wet habitat (Burns, 2004); and flooding with a water depth of 2 cm as the aquatic habitat. Each treatment set consisted of 8 replicates, and the plants subjected to three treatments were arrayed randomly. The initial dry biomasses of seedlings before treatments were determined on control groups (72°C, dry for 48 hr). After 8 weeks of water treatments, the total root length, root surface area, root volume, and root tip number were determined with the WinRHIZO Root Analysis System (Version 3.9, Regent Instruments Inc.). The total leaf area of *M. micrantha* plants was measured with a leaf area meter (Yaxin‐1241, Yaxinliji Technology Co., Ltd.). The roots, stems, and leaves of *M. micrantha* were dried (72°C, 48 hr), and their biomasses were determined. In addition, the relative growth rate (the averaged increase in dry matter per unit of dry mass per day over 56 days; Lombardi & Sebastiani, 2005), specific leaf area (leaf area per unit of leaf dry mass; Evans & Poorter, 2010), and root/shoot ratio of *M. micrantha* were calculated.

### 2.4 Survival of *M. micrantha* at different water depths

The experiment was conducted in Guangzhou (E 113°21′, N 23°9′, elevation 14 m), Guangdong Province, China from April to May in 2016. Four seedlings at the 2‐leaf stage were transplanted to one plastic pot with a diameter of 12 cm containing sandy soil. Flooding treatments were applied when the *M. micrantha* seedlings were at the 4‐leaf stage with heights of ~6 cm. The tested flooding depths were 0, 2, 4, 6, and 8 cm. The *M. micrantha* seedlings were planted in soil with a 90% water‐holding capacity as the control treatment. To calculate the survival rate, each treatment had eight replicates, each with 32 plants. Seedlings that grew above the water surface were regarded as surviving plants (preliminary experiments showed that *M. micrantha* would survive as long as it grew above the water surface). When all the *M. micrantha* seedlings underwater had died (after 4 weeks), the experiment was terminated, and the survival rates of *M. micrantha* at different water depths were calculated.

### 2.5 Statistical analysis

**Figure 2:**
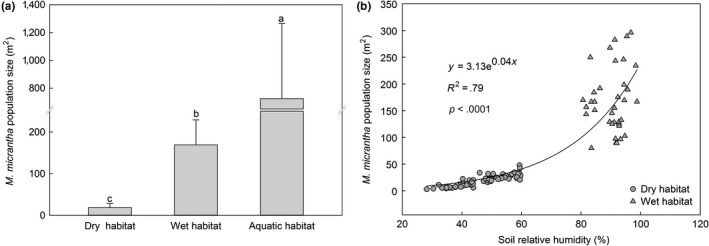
Difference in population size of *Mikania micrantha* in drylands (dry habitats; *n* = 74), on riverbanks (wet habitats; *n* = 36), and in wetlands with shallow water (aquatic habitats; *n* = 52; mean ± *SD*). (a) Different letters above the bar indicate significant differences at *p* < .05 according to the results of a post hoc Tukey's test. Degree of freedom (*df*) (between groups) = 2; *df* (within groups) = 159; *df* (total) = 161. (b) There were 74 of *Mikania micrantha* populations in dry habitats (*n* = 74) and 36 in wet habitats (*n* = 36)

**Figure 3:**
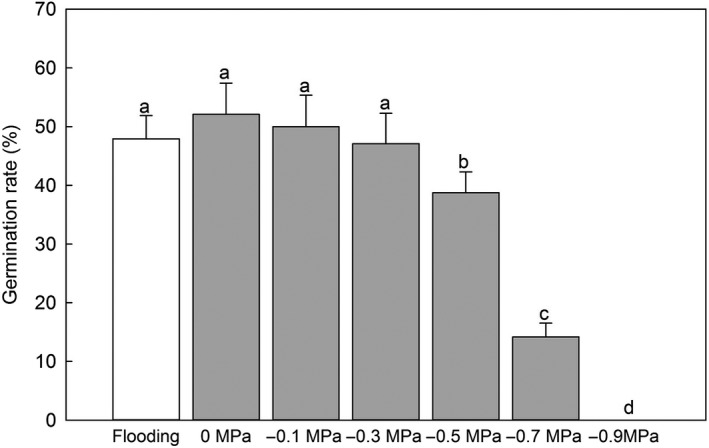
Effects of flooding and osmotic potential on the germination of *Mikania micrantha* after 14 days (mean ± *SD*, *n* = 8). Different letters above the bar indicate significant differences at *p* < .05 according to the results of a post hoc Tukey's test. Degree of freedom (*df*) (between groups) = 6; *df* (within groups) = 49; *df* (total) = 55

**Figure 4:**
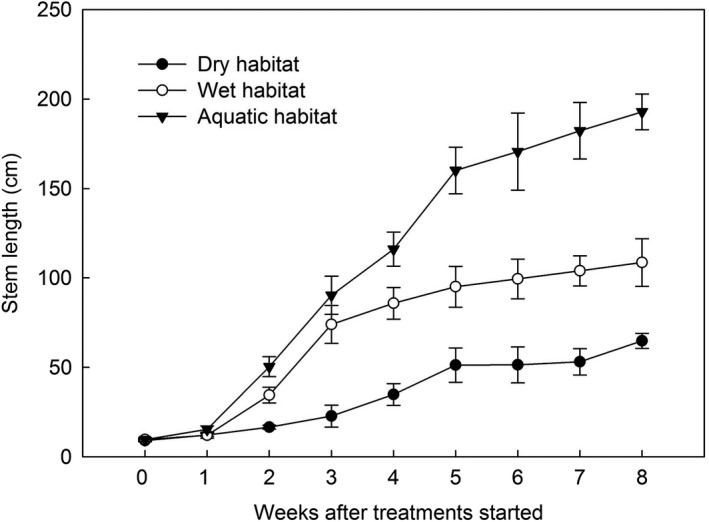
Main stem length of *Mikania micrantha* seedlings in the dry habitat (soil with a 40% water‐holding capacity), wet habitat (soil with a 90% water‐holding capacity), and aquatic habitat (flooding with a water depth of 2 cm; mean ± *SE*, *n* = 8). Degree of freedom (*df*) (between groups) = 6; *df* (within groups) = 49; *df* (total) = 55

All statistical tests were performed using SPSS 16.0 (SPSS Inc.). The population size, germination rate, growth index, and root morphology of *M. micrantha* under different water conditions were compared using a one‐way ANOVA followed by Tukey's test at *p* < .05. All observations were independent of one another, and the scores in groups were normally distributed. A univariate *F* test for each variable was used to interpret the respective effects. The equality of error variances was tested by using Levene's test, and the error variance of the dependent variable was considered to be equal across groups when *p* > .05. A linear regression analysis was used to assess the response of the population size of *M. micrantha* (as the dependent variable) to relative soil humidity (as the independent variable). Figure 1 was drawn using ArcGIS 10.2 software (ESRI) and Figures 2, 3, 4 were drawn using SigmaPlot 12.5 software (Systat Software Inc.).

## 3 RESULTS

### 3.1 *M. micrantha* population sizes in different habitats

The results showed that the *M. micrantha* population size in the aquatic habitat was the largest, followed by wet habitat, and the smallest one was found in dry habitat. The *M. micrantha* population size in the aquatic habitat was 3 and 38 times larger than that in the wet and dry habitats, respectively (Figure 2a). The population size in the dry and wet habitats depended on the soil moisture content (*y* = 3.13e^0.04^ *^x^*, *R* ^2^ = .79, *p* < .0001; Figure 2b).

### 3.2 Germination rate and seedling growth under different water conditions

In the seed germination test, the seeds of *M. micrantha* could germinate underwater (flooding). The germination rate was not significantly different between the flooding and the lower osmotic potential treatments (0 to −0.3 MPa). With an increase in the osmotic potential, the seed germination rate decreased significantly (Figure 3).

In the water‐submerging test, the speed at which the main stem of *M. micrantha* extended varied significantly among the three water environments (Figure 4). *M. micrantha* stems extended most rapidly in the aquatic habitat (flooding with a water depth of 2 cm), most slowly in the dry habitat (soil with 40% water‐holding capacity), and at an intermediate rate in the wet habitat (soil with 90% water‐holding capacity). Eight weeks after the treatments, the main stem length in the aquatic habitat was 2 times longer than that in the wet habitat and 3 times longer than that in the dry habitat.

After 8 weeks of different water treatments, the total biomass of *M. micrantha* in the aquatic and wet habitats was much higher than that in the dry habitat (Table 1). The relative growth rate showed a similar trend (Table 1).

**Table 1:** Growth index of *Mikania micrantha* seedlings in a dry habitat (soil with a 40% water‐holding capacity), wet habitat (soil with a 90% water‐holding capacity), and aquatic habitat (flooding with a water depth of 2 cm; mean ± *SD*, *n* = 8)

Variables/Habitats	Dry habitat	Wet habitat	Aquatic habitat
Total biomass (g)	2.67 ± 0.11b	7.06 ± 0.14a	7.07 ± 0.17a
Relative growth rate (mg g^−1^ d^−1^)	68.85 ± 0.73b	86.22 ± 0.37a	86.24 ± 0.43a
Root biomass (g)	0.49 ± 0.02b	1.22 ± 0.04a	1.18 ± 0.04a
Stem biomass (g)	1.10 ± 0.06c	3.34 ± 0.13b	4.34 ± 0.22a
Leaf biomass (g)	1.08 ± 0.09c	2.49 ± 0.13a	1.55 ± 0.22b
Root/shoot ratio	0.23 ± 0.01a	0.21 ± 0.01b	0.20 ± 0.01b
Total leaf area (cm^2^)	255.46 ± 17.44c	725.48 ± 39.12a	356.76 ± 48.09b
Specific leaf area (cm^2^/g)	237.73 ± 15.07b	290.96 ± 7.61a	230.90 ± 11.41b
Branch number	6.00 ± 0.93b	11.00 ± 0.93a	4.88 ± 0.83b
Stem node length (cm)	3.72 ± 0.30c	8.64 ± 0.73b	17.68 ± 1.71a

Note

Data with different letters within the same line indicate significant differences at *p* < .05 according to the results of a post hoc Tukey's test. Degrees of freedom (*df*) (between groups) = 2; *df* (within groups) = 21; *df* (total) = 23.

The root biomass in the aquatic habitat was similar to that in the wet habitat, and both were significantly higher than that in the dry habitat (*p* < .05). Compared with the wet habitat, the aquatic habitat significantly promoted the stem biomass of *M. micrantha* while the dry habitat significantly inhibited it (*p* < .05). The leaf biomass of *M. micrantha* in the aquatic and dry habitats was significantly lower than that in the wet habitat, while the leaf biomass in the aquatic habitat was significantly higher than that in the dry habitat (*p* < .05; Table 1).

The root/shoot ratio of *M. micrantha* in the dry habitat was the highest among the three treatments and was significantly higher than that in the wet and aquatic habitats (*p* < .05). The root/shoot ratio in the aquatic habitat was similar to that in the wet habitat. Compared with the wet habitat, the aquatic and dry habitats inhibited the leaf area of *M. micrantha* (*p* < .05), resulting in areas that were 51% and 65% smaller compared with the wet habitat, respectively. The specific leaf area and branch number of *M. micrantha* in the aquatic and dry habitats were less than those in the wet habitat (*p* < .05). Compared with the wet habitat, the aquatic habitat promoted the stem node length while the dry habitat inhibited it (*p* < .05; Table 1).

Compared with the wet habitat, *M. micrantha* in the aquatic habitat showed significantly enhanced total root length, root surface area, root volume, and root tip number, whereas the dry habitat significantly inhibited these parameters (*p* < .05; Table 2).

**Table 2:** Root morphological changes in *Mikania micrantha* in a dry habitat (soil with a 40% water‐holding capacity), wet habitat (soil with a 90% water‐holding capacity), and aquatic habitat (flooding with a water depth of 2 cm; mean ± *SD*, *n* = 8)

Treatment	Total root length (cm)	Root surf. area (cm^2^)	Root volume (cm^3^)	Root tip number
Dry habitat	735.62 ± 17.18c	98.80 ± 17.09c	2.21 ± 0.12c	774.38 ± 25.12c
Wet habitat	1,877.06 ± 59.78b	450.35 ± 7.97b	8.55 ± 0.37b	1,274.13 ± 44.93b
Aquatic habitat	3,751.52 ± 78.94a	761.97 ± 16.79a	13.33 ± 0.45a	3,234.88 ± 101.17a

Note

Data with different letters within the same column indicate significant differences at *p* < .05 according to the results of a post hoc Tukey's test. Degrees of freedom (*df*) (between groups) = 2; *df* (within groups) = 21; *df* (total) = 23.

### 3.3 Survival rate of *M. micrantha* seedlings at different water depths

**Figure 5:**
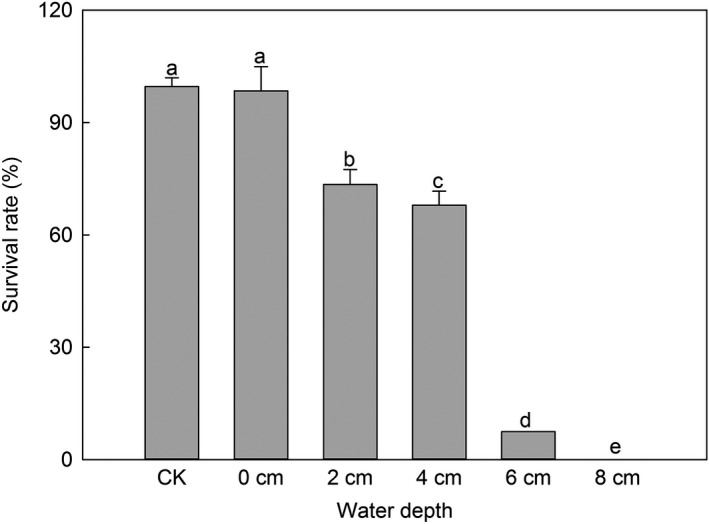
Survival rates of *Mikania micrantha* seedlings at different water depths (means ± *SD*, *n* = 8). In the CK, the seedlings were planted in soil with a 90% water‐holding capacity. Different letters above the bar indicate significant differences at *p* < .05 according to the results of a post hoc Tukey's test. Degree of freedom (*df*) (between groups) = 5; *df* (within groups) = 42; *df* (total) = 47

Almost all *M. micrantha* seedlings could survive at a water depth of 0 cm. The survival rate at a water depth of 0 cm was similar to that in the wet habitat (soil with 90% water‐holding capacity). With an increase in water depth, the survival rate decreased significantly (*p* < .05). When the water depth was 6 cm, the survival rate was only 7.4%. None of the *M. micrantha* plants survived at a water depth of 8 cm (Figure 5).

## 4 DISCUSSION

Water is an important environmental factor that dictates the growth and distribution of plants (Boyer, 1982; Kelly & Goulden, 2008). Therefore, the invasion process of exotic plants is partially dependent on the soil moisture (Kercher & Zedler, 2004; Sher, Marshall, & Gilbert, 2000). In this study, the largest population size of *M. micrantha* was found in the aquatic habitat in the field (Figure 2a), and the controlling experiment also showed that flooding could significantly promote the vine stem growth of *M. micrantha* (Figure 4). Our results were supported by previous studies that indicated that *M. micrantha* prefers wetlands and can even cover large areas of ponds (Zhang et al., 2004). Therefore, *M. micrantha* can survive in shallow water and the elongation of its vine stems can be stimulated by flooding. Similar studies elsewhere have found that other invasive species (e.g., *Bidens pilosa* and *Alternanthera philoxeroides*) are also very tolerant to waterlogging (Chen et al., 2013; Yue et al., 2019). In addition, the results of this study also indicated that the stem growth of *M. micrantha* was very sensitive to drought (Figure 4). Although studies have shown that *M. micrantha* exhibits broad niche breadth under drought stress (Shen et al., 2015; Zhou, Liu, Sun, Wu, & Hou, 2015), our results indicated that the growth of *M. micrantha* was significantly inhibited under drought conditions, presumably drought habitats is not susceptible to *M. micrantha* invasion. These findings suggest that flooding with shallow water would promote the invasiveness of *M. micrantha* while drought would be a limiting factor for its growth.

Since *M. micrantha* prefers aquatic habits, it will meet the demands of flooding in places where water is abundant and may therefore have gradually developed characteristics to adapt to flooded environments. Internode and stem elongation are related to the enhancement of oxygen transport and dispersal in plants (Fu, Xu, Ronald, & Bailey, 2006; Kawano, Ito, & Sakagami, 2008), and plants can accelerate the elongation of stems to quickly “escape” the adverse conditions of flooding (Groeneveld & Voesenek, 2003; Voesenek et al., 2004). The main stem length and stem node length of *M. micrantha* increased significantly in the aquatic habitat in this study (Table 1 and Figure 4). These morphological changes contribute to a better adaptation of *M. micrantha* to aquatic environments or flooding tolerance. Thus, the rapid growth of stems in shallow water might help *M. micrantha* to occupy the habitats and favor its invasion. In addition, we found that the total root length, root surface area, root volume, and root tip number of *M. micrantha* were all significantly increased in the aquatic habitat (Table 2). This observation may be related to flooding, which could induce the adventitious root primordia to form adventitious roots, while the ventilated tissue in the adventitious roots expanded the root porosity to increase the ability to obtain and transport oxygen (Drew, Jackson, & Giffard, 1979; Visser, Colmer, Blom, & Voesenek, 2000). Flood‐tolerant plants are often able to form a large number of adventitious roots in the aquatic environments to adapt to hypoxic habitats (Ayi et al., 2016; Dawood et al., 2014). Therefore, for *M. micrantha*, the growth of a large number of adventitious roots is favorable for its adaptation to aquatic environments.

The *M. micrantha* population sizes on both sides of rivers did not represent the largest population among the three examined water conditions, although the soil water content was high at the riverbanks. This finding may be related to the inability of *M. micrantha* to survive in deep water. Our results showed that *M. micrantha* can grow at a water depth of 6 cm with a low survival rate but can't survive at a water depth of 8 cm (Figure 5). Similarly, Xu et al. (2013) found that the nutritional propagation of *M. micrantha* could not continue at a water depth >6 cm. Precipitation is high in southern China (more than 1,000 mm) and concentrated from April to September (Yu, 1996); therefore, the water levels of the main rivers are often substantially increased in the rainy season. If the water levels rise too high and cover *M. micrantha* by more than 8 cm, it will be fatal to the plant. In South China, the rainy season often deepens the river, which is unfavorable for *M. micrantha* growing along the riverbank.

In the agroecosystem, the aquatic and wet habitats should be eliminated around the farmland and orchard sits since these two types of habitats are more conducive to the growth of *M. micrantha*. Because the vine stems of *M. micrantha* can grow infinitely as long as there is adequate water, we should prioritize the management of *M. micrantha* populations in aquatic and wet habitats with shallow water or riverbanks over those in terrestrial habitats.

In conclusion, *M. micrantha* prefers aquatic habitats and flooding with shallow water promoted its stem elongation, which makes *M. micrantha* form largest population size under waterlogging conditions. Thus, flooding with shallow water could be conducive to the invasiveness of *M. micrantha*, which implies that water is the key determinant during the intrusion process of *M. micrantha* populations. Future research is needed to do with regard to the ecological adaptability mechanism of *M. micrantha* to waterlogging.

## CONFLICTS OF INTEREST

None declared.

## AUTHOR CONTRIBUTIONS

MY and XT designed the study; MY and WL wrote the manuscript; AY conducted most of the experimental work; HY and CY analyzed the data; and all co‐authors contributed to writing the manuscript and discussions.

## Data Availability

All the data used in this manuscript are available from https://osf.io/a5ymf/.

## References

[ece35465-bib-0001] Awada, T. , Skolaut, K. , Battipaglia, G. , Saurer, M. , Riveros‐Iregui, D. A. , Schapaugh, A. , … Cherubini, P. (2019). Tree‐ring stable isotopes show different ecophysiological strategies in native and invasive woody species of a semiarid riparian ecosystem in the Great Plains of the United States. Ecohydrology, 12(3), e2074 10.1002/eco.2074

[ece35465-bib-0002] Ayi, Q. , Zeng, B. , Liu, J. , Li, S. , Van Bodegom, P. M. , & Cornelissen, J. H. C. (2016). Oxygen absorption by adventitious roots promotes the survival of completely submerged terrestrial plants. Annals of Botany, 118(4), 675–683. 10.1093/aob/mcw051 PMC5055620 27063366

[ece35465-bib-0003] Boyer, J. S. (1982). Plant productivity and environment. Science, 218(4571), 443–448. 10.1126/science.218.4571.443 17808529

[ece35465-bib-0004] Burns, J. H. (2004). A comparison of invasive and non‐invasive dayflowers (Commelinaceae) across experimental nutrient and water gradients. Diversity and Distributions, 10(5–6), 387–397. 10.1111/j.1366-9516.2004.00105.x

[ece35465-bib-0005] Chen, Y. , Zhou, Y. , Yin, T. F. , Liu, C. X. , & Luo, F. L. (2013). The invasive wetland plant *Alternanthera philoxeroides* shows a higher tolerance to waterlogging than its native congener *Alternanthera sessilis* . PLoS ONE, 8(11), e81456 10.1371/journal.pone.0081456 24303048 PMC3841148

[ece35465-bib-0006] Chytrý, M. , Wild, J. , Pyšek, P. , Jarošík, V. , Dendoncker, N. , Reginster, I. , … Settele, J. (2012). Projecting trends in plant invasions in Europe under different scenarios of future land‐use change. Global Ecology and Biogeography, 21(1), 75–87. 10.1111/j.1466-8238.2010.00573.x

[ece35465-bib-0007] Dawood, T. , Rieu, I. , Wolters‐Arts, M. , Derksen, E. B. , Mariani, C. , & Visser, E. J. W. (2014). Rapid flooding‐induced adventitious root development from preformed primordia in *Solanum dulcamara* . AoB Plants, 6, 1–13. 10.1093/aobpla/plt058 PMC3922303 24790121

[ece35465-bib-0008] Day, M. D. , Clements, D. R. , Gile, C. , Senaratne, W. K. A. D. , Shen, S. , Weston, L. A. , & Zhang, F. (2016). Biology and impacts of pacific islands invasive species. 13. *Mikania micrantha* kunth (Asteraceae). Pacific Science, 70(3), 257–285. 10.2984/70.3.1

[ece35465-bib-0009] Dong, X. , Patton, J. , Wang, G. , Nyren, P. , & Peterson, P. (2014). Effect of drought on biomass allocation in two invasive and two native grass species dominating the mixed‐grass prairie. Grass and Forage Science, 69(1), 160–166. 10.1111/gfs.12020

[ece35465-bib-0010] Drew, M. C. , Jackson, M. B. , & Giffard, S. J. P. (1979). Ethylene‐promoted adventitious rooting and development of cortical air spaces (aerenchyma) in roots may be adaptive responses to flooding in *Zea mays* L. Planta, 147(1), 83–88. 10.1007/bf00384595 24310899

[ece35465-bib-0011] Du, F. , Yang, Y. M. , Li, J. Q. , & Yin, W. Y. (2006). A review of *Mikania* and the impact of *M. micrantha* (Asteraceae) in Yunnan. Acta Botanica Yunnanica, 28(5), 505–508. 10.3969/j.issn.2095-0845.2006.05.011

[ece35465-bib-0012] Ensslin, A. , Mollel, N. P. , Hemp, A. , & Fischer, M. (2018). Elevational transplantation suggests different responses of African submontane and savanna plants to climate warming. Journal of Ecology, 106(1), 296–305. 10.1111/1365-2745.12842

[ece35465-bib-0013] Evans, J. R. , & Poorter, H. (2010). Photosynthetic acclimation of plants to growth irradiance: The relative importance of specific leaf area and nitrogen partitioning in maximizing carbon gain. Plant, Cell & Environment, 24(8), 755–767. 10.1046/j.1365-3040.2001.00724.x

[ece35465-bib-0014] Fu, K. T. , Xu, K. , Ronald, P. C. , & Bailey, S. J. (2006). A variable cluster of ethylene response factor‐like genes regulates metabolic and developmental acclimation responses to submergence in rice. Plant Cell, 18(8), 2021–2034. 10.1105/tpc.106.043000 16816135 PMC1533987

[ece35465-bib-0015] Groeneveld, H. W. , & Voesenek, L. A. C. J. (2003). Submergence‐induced petiole elongation in *Rumex palustris* is controlled by developmental stage and storage compounds. Plant and Soil, 253(1), 115–123. 10.1023/a:1024511232626

[ece35465-bib-0016] Holm, L. G. , Plucknett, D. L. , Pancho, J. V. , & Herberger, J. P. (1977). The world's worst weeds: Distribution and biology. Honolulu, HI: University Press of Hawaii.

[ece35465-bib-0017] Huang, Z. L. , Cao, H. L. , Liang, X. D. , Ye, W. H. , Feng, H. L. , & Cai, C. X. (2000). The growth and damaging effect of *Mikania micrantha* indifferent habitats. Journal of Tropical and Subtropical Botany, 8(2), 131–138. 10.3969/j.issn.1005-3395.2000.2.009

[ece35465-bib-0018] Karkanis, A. , Ntatsi, G. , Alemardan, A. , Petropoulos, S. , & Bilalis, D. (2019). Interference of weeds in vegetable crop cultivation, in the changing climate of Southern Europe with emphasis on drought and elevated temperatures: A review. Journal of Agricultural Science, 156(10), 1175–1185. 10.1017/S0021859619000108

[ece35465-bib-0019] Kawano, N. , Ito, O. , & Sakagami, J. I. (2008). Relationship between shoot elongation and dry matter weight during submergence in *Oryza sativa* L. and *O. glaberrima* Steud. rice cultivars. Plant Production Science, 11(3), 316–323. 10.1626/pps.11.316

[ece35465-bib-0020] Kelly, A. E. , & Goulden, M. L. (2008). Rapid shifts in plant distribution with recent climate change. Proceedings of the National Academy of Sciences, 105(33), 11823–11826. 10.1073/pnas.0802891105 PMC2575286 18697941

[ece35465-bib-0021] Kercher, S. M. , & Zedler, J. B. (2004). Flood tolerance in wetland angiosperms: A comparison of invasive and noninvasive species. Aquatic Botany, 80(2), 89–102. 10.1016/j.aquabot.2004.08.003

[ece35465-bib-0022] Klemedtsson, L. , Svensson, B. H. , & Rosswall, T. J. B. (1988). Relationship between soil moisture content and nitrous oxide production during nitrification and denitrification. Biology and Fertility of Soils, 6(2), 106–111. 10.1007/bf00257658

[ece35465-bib-0023] Lombardi, L. , & Sebastiani, L. (2005). Copper toxicity in *Prunus cerasifera*: Growth and antioxidant enzymes responses of in vitro grown plants. Plant Science, 168(3), 797–802. 10.1016/j.plantsci.2004.10.012

[ece35465-bib-0024] Lowe, S. , Browne, M. , Boudjelans, S. , & De Poorter, M. (2001). 100 of the world's worst invasive alien apecies: A selection from the global invasive species database. Auckland, New Zealand: Invasive Species Specialist Group (ISSG).

[ece35465-bib-0025] Moore, G. A. (2001). Soil guide: A handbook for understanding and managing agricultural soils. Perth, WA, Australia: National Landcare and Department of Agriculture Western Australia.

[ece35465-bib-0026] Murphy, S. T. , Subedi, N. , Jnawali, S. R. , Lamichhane, B. R. , Upadhyay, G. P. , Kock, R. , & Amin, R. (2013). Invasive *Mikania* in Chitwan National Park, Nepal: The threat to the greater one‐horned rhinoceros *Rhinoceros unicornis* and factors driving the invasion. Oryx, 47(3), 361–368. 10.1017/S003060531200124X

[ece35465-bib-0027] Ozaslan, C. , Farooq, S. , Onen, H. , Bukun, B. , Ozcan, S. , & Gunal, H. (2016). Invasion potential of two tropical *Physalis* species in arid and semi‐arid climates: Effect of water‐salinity stress and soil types on growth and fecundity. PLoS ONE, 11(10), e0164369 10.1371/journal.pone.0164369 27741269 PMC5065205

[ece35465-bib-0028] Pejchar, L. , & Mooney, H. A. (2009). Invasive species, ecosystem services and human well‐being. Trends in Ecology & Evolution, 24(9), 497–504. 10.1016/j.tree.2009.03.016 19577817

[ece35465-bib-0029] Saptiningsih, E. , Dewi, K. , Santosa, S. , & Purwestri, Y. A. (2018). Clonal integration of the invasive plant *Wedelia trilobata* (L.) Hitch in stress of flooding type combination. International Journal of Plant Biology, 9(1), 30–47. 10.4081/pb.2018.7526

[ece35465-bib-0030] Shen, S. C. , Xu, G. F. , Clements, D. R. , Jin, G. M. , Chen, A. D. , Zhang, F. D. , & Kato‐Noguchi, H. (2015). Suppression of the invasive plant mile‐a‐minute (*Mikania micrantha*) by local crop sweet potato (*Ipomoea batatas*) by means of higher growth rate and competition for soil nutrients. BMC Ecology, 15(1), 2–10. 10.1186/s12898-014-0033-5 25626963 PMC4325956

[ece35465-bib-0031] Sher, A. A. , Marshall, D. L. , & Gilbert, S. A. (2000). Competition between native *Populus deltoides* and invasive *Tamarix ramosissima* and the implications for reestablishing flooding disturbance. Conservation Biology, 14(6), 1744–1754. 10.2307/2641526 35701936

[ece35465-bib-0032] Turner, K. G. , Nurkowski, K. A. , & Rieseberg, L. H. (2017). Gene expression and drought response in an invasive thistle. Biological Invasions, 19(3), 875–893. 10.1007/s10530-016-1308-x

[ece35465-bib-0033] Visser, E. J. W. , Colmer, T. D. , Blom, C. W. P. M. , & Voesenek, L. A. C. J. (2000). Changes in growth, porosity, and radial oxygen loss from adventitious roots of selected mono‐ and dicotyledonous wetland species with contrasting types of aerenchyma. Plant, Cell & Environment, 23(11), 1237–1245. 10.1046/j.1365-3040.2000.00628.x

[ece35465-bib-0034] Voesenek, L. A. C. J. , Rijnders, J. H. G. M. , Peeters, A. J. M. , Van, D. E. , Steeg, H. M. , & Dekroon, H. (2004). Plant hormones regulate fast shoot elongation under water: From genes to communities. Ecology, 85(1), 16–27. 10.1890/02-740

[ece35465-bib-0036] Waterhouse, D. F. (1994). Biological control of weeds: Southeast Asian prospects. Canberra, Australia: ACIAR.

[ece35465-bib-0037] Wei, S. , Zhang, C. , Li, X. , Cui, H. , Huang, H. , Sui, B. , … Zhang, H. (2009). Factors affecting buffalobur (*Solanum rostratum*) seed germination and seedling emergence. Weed Science, 57(5), 521–525. 10.1614/we-09-054.1

[ece35465-bib-0038] Xu, G. F. , Shen, S. C. , Zhang, F. D. , Li, T. L. , & Zhang, Y. H. (2013). Effect of soil‐water conditions on survival rate and morphological plasticity of clonal plant *Mikania micrantha* H B K. Scientia Agricultura Sinica, 46(15), 3134–3141. 10.3864/j.issn.0578-1752.2013.15.007

[ece35465-bib-0039] Yu, G. (1996). Climatic characteristics of precipitation over South China in recent 40 Years. Journal of Tropical Meteorology, 12(3), 252–256.

[ece35465-bib-0040] Yue, M. , Shen, H. , Li, W. , Chen, J. , Ye, W. , Tian, X. , … Cheng, S. (2019). Waterlogging tolerance of *Bidens pilosa* translates to increased competitiveness compared to native *Bidens biternata* . Plant and Soil, 437(1–2), 301–311. 10.1007/s11104-019-03967-5

[ece35465-bib-0041] Zhang, L. Y. , Ye, W. H. , Cao, H. L. , & Feng, H. L. (2004). *Mikania micrantha* H.B.K. in China – An overview. Weed Research, 44(1), 42–49. 10.1111/j.1365-3180.2003.00371.x

[ece35465-bib-0042] Zhou, S. , Liu, N. , Sun, Y. , Wu, D. , & Hou, Y. (2015). MmZFP1 response to abiotic stress in the invasive plant *Mikania micrantha* . Nature Environment and Pollution Technology, 14(2), 251–258.

